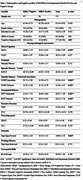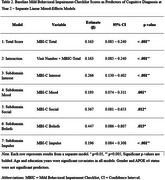# Unveiling Cognitive Decline Through the Lens of Mild Behavioral Impairment: Longitudinal Insights from the Southeast Asia BIOCIS Study

**DOI:** 10.1002/alz70857_106254

**Published:** 2025-12-25

**Authors:** Yi Jin Leow, Pricilia Tanoto, Nagaendran Kandiah

**Affiliations:** ^1^ nil, nil, nil, Nicaragua; ^2^ Dementia Research Centre (Singapore), Lee Kong Chian School of Medicine, Nanyang Technological University, Singapore, Singapore; ^3^ Lee Kong Chian School of Medicine, Nanyang Technological University, Singapore, Singapore

## Abstract

**Background:**

Subtle behavioral changes may signal early cognitive decline, presenting opportunities for intervention. Mild Behavioral Impairment (MBI) is recognized as a precursor to cognitive decline and Alzheimer's disease (AD). The MBI‐Checklist (MBI‐C) has been validated as an effective tool for detecting MBI and assessing neuropsychiatric symptoms. However, its predictive value remains underexplored in Southeast Asian populations. Cultural factors may contribute to underreporting of emotional and behavioral symptoms, making objective assessment crucial. This study evaluates whether MBI‐C scores, assessed longitudinally, predict cognitive outcomes.

**Method:**

The study included 571 participants (mean age:61.43±9.82years;44%male) from the Biomarkers and Cognition Study, Singapore (BIOCIS), assessed over two visits, one year apart. Participants were classified as cognitively normal, subjective cognitive decline, mild cognitive impairment, or mild dementia based on established criteria. Behavioral symptoms were measured using the self‐reported MBI‐C with a clinical cutoff of 5.5. Longitudinal cognitive outcomes were analyzed using linear mixed‐effects models, incorporating MBI‐C total and subdomain scores at Visit 1 as predictors. Covariates included age, gender, education years, and APOEε4 status. A Visit×MBI‐C interaction term assessed temporal effects. Random intercepts captured individual variability.

**Result:**

At Visit 1, participants with clinical MBI‐C scores demonstrated significantly poorer performance in global cognition (*p* = .035), verbal episodic memory (*p* = .020), verbal and associative episodic memory (*p* = .005 for immediate‐recall, *p* = .010 for delayed‐recall), visuospatial memory (*p* = .001 for immediate‐recall, *p* = .019 for delayed‐recall), and attention (*p* = .010). Higher MBI‐C total scores at Visit 1 significantly predicted worse cognitive diagnoses at Visit 2(β=0.163, *p* < .001). The Visit×MBI‐C interaction (β=0.163, *p* < .001) suggested an intensifying relationship between MBI‐C scores and cognitive outcomes over time. Subdomain analyses revealed significant associations between cognitive decline and MBI‐C domains, including Interest (β=0.266, *p* < .001), Mood (β=0.193, *p* = .001), Social (β=0.367, *p* = .012), Abnormal Beliefs (β=0.447, *p* = .015), and Impulse Control (β=0.196, *p* < .001).

**Conclusion:**

Self‐reported MBI‐C scores predict early cognitive decline, reinforcing MBI symptoms as clinical markers. The strengthening association over time underscores their progressive impact on cognition. Notably, the Abnormal Beliefs subdomain showed the strongest association with cognitive deterioration, suggesting its utility as a marker for progression. Addressing MBI could mitigate cognitive decline, emphasizing its role in early intervention. Furthermore, the tendency in Southeast Asia to underreport behavioral symptoms may indicate that observed associations reflect more severe underlying pathology, reinforcing the robustness of these findings.